# Supplementary material for: Medical Cost of Cancer Care for Privately Insured Children in Chile
Source: Int J Environ Res Public Health. 2021 Jun 23;18(13):6746. doi: 10.3390/ijerph18136746 (PMC8267683; doi:10.3390/ijerph18136746)
Supplement: Supplementary file 1 [file ijerph-18-06746-s001.zip › ijerph-1238850-supplementary.pdf]

Supplementary Material for:

Medical Cost of Cancer Care for Privately Insured Children in Chile,

by Florencia Borrescio-Higa and Nieves Valdés

Table S1. Quasi-Maximum likelihood estimates of Generalized Linear Models with log link function and gamma distribution for total medical costs and out-of-pocket payments, by type of care, 2007 to 2018. Private health insurance system.

	**Total Costs**			**Out-of-pocket**		
	(1)	(2)	(3)	(4)	(5)	(6)
VARIABLES	All	Inpatient	Outpatient	All	Inpatient	Outpatient
Patient						
Age 5-9	-0.429***	-0.464***	0.041	-0.462***	-0.528***	-0.069
	(0.005)	(0.006)	(0.006)	(0.008)	(0.010)	(0.009)
Age 10-14	-0.297***	-0.34***	0.22**	-0.222*	-0.272**	0.039
	(0.007)	(0.008)	(0.010)	(0.014)	(0.017)	(0.009)
Age 15-17	-0.297***	-0.35***	0.279**	-0.215*	-0.273**	0.061
	(0.008)	(0.010)	(0.012)	(0.015)	(0.018)	(0.010)
Male	0.042	0.043	0.04	-0.026	-0.038	0.014
	(0.003)	(0.003)	(0.005)	(0.006)	(0.007)	(0.005)
Leukemia	0.922***	0.945***	0.609***	0.672***	0.722***	0.354***
	(0.004)	(0.005)	(0.006)	(0.009)	(0.011)	(0.006)
Brain or nervous system	0.687***	0.68***	0.688***	0.837***	0.841***	0.738***
	(0.007)	(0.008)	(0.012)	(0.016)	(0.017)	(0.022)
Non-Hodgkin's lymphoma	0.613***	0.634***	0.393***	0.514***	0.523***	0.517***
	(0.014)	(0.016)	(0.015)	(0.023)	(0.027)	(0.022)
Bone or articular cartilage	0.659***	0.682***	0.344**	0.638***	0.691***	0.355***
	(0.015)	(0.016)	(0.018)	(0.033)	(0.038)	(0.015)
Mesothelioma soft tissues	0.399***	0.403***	0.237	-0.18	-0.224	0.05
	(0.018)	(0.020)	(0.039)	(0.022)	(0.028)	(0.016)
GES payment	0.273***	0.277***	0.158**	-0.484***	-0.512***	-0.287***
	(0.00)	(0.003)	(0.004)	(0.01)	(0.007)	(0.005)
Policyholder						
Residence in Metropolitan region	0.012	0.009	0.059*	0.058	0.061	0.051
	(0.00)	(0.001)	(0.001)	(0.00)	(0.002)	(0.001)
Q1 of annual income	0	0	-0.001*	-0.001*	-0.001	-0.001
	(0.00)	(0.000)	(0.000)	(0.00)	(0.000)	(0.000)
Q2 of annual income	-0.029	-0.023	-0.06	-0.032	-0.01	-0.16**
	(0.00)	(0.004)	(0.005)	(0.01)	(0.009)	(0.005)
Q3 of annual income	0.099*	0.13**	-0.227***	0.129	0.143	0.08
	(0.00)	(0.004)	(0.006)	(0.01)	(0.008)	(0.005)
Q4 of annual income	0.022	0.056	-0.359***	-0.124	-0.05	-0.602***
	(0.01)	(0.010)	(0.014)	(0.02)	(0.025)	(0.016)
Policyholder's demographic controls	Yes	Yes	Yes	Yes	Yes	Yes
Year dummies	Yes	Yes	Yes	Yes	Yes	Yes
Observations	3 644	3 644	3 644	3 644	3 644	3 644

Note: Robust standard errors in parentheses, clustered at the policy holder's id level. *** p<0.01, ** p<0.05, * p<0.1. The reference category for type of cancer is “other cancer” diagnosis. Abbreviations: GES, Garantías Explícitas de Salud; Q1, Q2, Q3, Q4, first, second, third, and fourth quintile of income distribution, respectively.
